# Supplementary material for: Histone Deacetylase Inhibitors Prevent Cytokine-Induced β Cell Dysfunction Through Restoration of Stromal Interaction Molecule 1 Expression and Activation of Store-Operated Calcium Entry
Source: bioRxiv. 2023 Dec 8:2023.12.06.570443. Preprint. [Version 1] doi: 10.1101/2023.12.06.570443 (PMC10723426; doi:10.1101/2023.12.06.570443)
Supplement: 1 [file NIHPP2023.12.06.570443V1-supplement-1.pdf]

Supplemental Figure 1
Representative membranes of phosphorylation array assay in mouse islets treated with cytokines in the presence or absence of NaB.

(A)

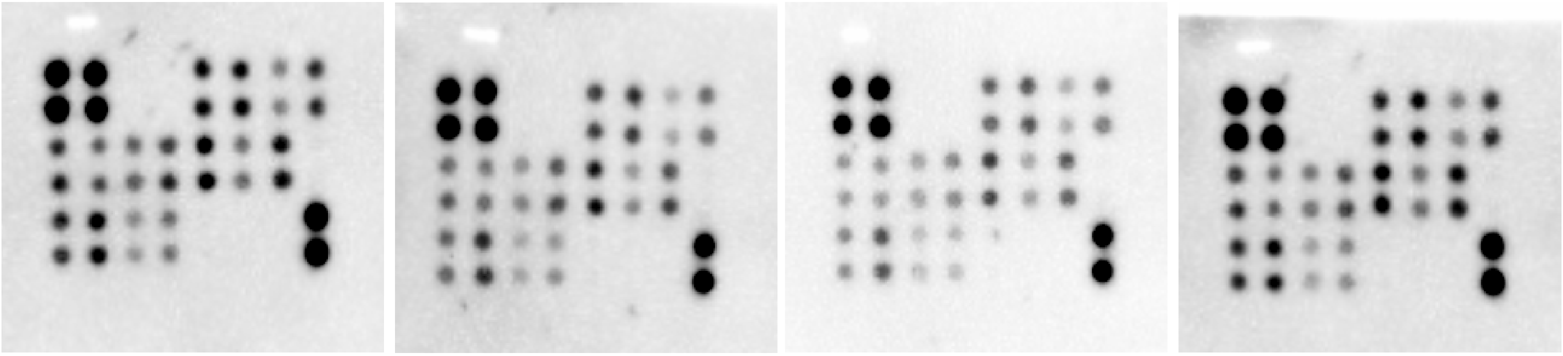

Cytokine	-	-	+	+
NaB	-	+	-	+

(B)

Each antibody is spotted in duplicate vertically

	A	B	C	D	E	F	G	H
1<br>2	POS	POS	NEG	NEG	Akt (P-S473)	CREB (P-S133)	ERK1 (P-T202/Y204) ERK2 (P-Y185/Y187)	GSK3a (P-S21)
3<br>4	GSK3b (P-S9)	HSP27 (P-S82)	JNK (P-T183)	MEK (P-S217/221)	MKK3 (P-S189)	MKK6 (P-S207)	MSK2 (P-S360)	mTOR (P-S2448)
5<br>6	p38 (P-T180/Y182)	P53 (P-S15)	P70S6K (P-T421/S424)	RSK1 (P-S380)	RSK2 (P-S386)	NEG	NEG	POS
